# Regulatory T cells in the bladder cancer tumor microenvironment: mechanisms of immune suppression and therapeutic opportunities

**DOI:** 10.3389/fmolb.2026.1898769

**Published:** 2026-08-10

**Authors:** Yusuke Fukiage, Nodoka Okubo, Minekatsu Taga, Yohei Sato, Naoki Terada

**Affiliations:** 1 Department of Urology, Faculty of Medical Sciences, University of Fukui, Fukui, Japan; 2 Department of Otorhinolaryngology, Head and Neck Surgery, Faculty of Medical Sciences, University of Fukui, Fukui, Japan; 3 Immunology and Allergy Research Unit, Department of Otorhinolaryngology, Head and Neck Surgery, Faculty of Medical Sciences, University of Fukui, Fukui, Japan

**Keywords:** *Bacillus* Calmette–Guérin (BCG), bladder cancer, immune checkpoint inhibitor, regulatory T cells (Tregs), tumor microenvironment

## Abstract

Bladder cancer is characterized by a highly dynamic tumor microenvironment (TME) that critically influences tumor progression, immune evasion, and therapeutic responsiveness. Among the immune populations in the TME, regulatory T cells (Tregs) play a central role in maintaining immune tolerance but also suppress effective antitumor immunity. Increasing evidence suggests that Tregs accumulate in bladder tumors and are associated with disease progression and reduced response to immunotherapies. The bladder cancer TME provides multiple signals that promote Treg recruitment, expansion, and functional stabilization, including chemokine-mediated trafficking, metabolic adaptation, and cytokine-driven differentiation. Interactions between Tregs and other microenvironmental components, such as cancer-associated fibroblasts, tumor-associated macrophages, endothelial cells, and extracellular matrix elements, further reinforce the immunosuppressive niche that facilitates tumor survival and therapy resistance. Recent advances in single-cell transcriptomics, spatial profiling, and multiomics analyses have revealed substantial heterogeneity among tumor-infiltrating Tregs, suggesting the existence of specialized subsets with distinct functional and metabolic properties in the bladder TME. These emerging insights highlight the importance of understanding Treg–TME crosstalk in shaping the immune landscape of bladder cancer. Targeting the mechanisms regulating Treg recruitment, stability, or suppressive function may represent a promising strategy to enhance the efficacy of immunotherapies including *Bacillus* Calmette–Guérin therapy. This review summarizes recent advances in Treg biology in bladder cancer and highlights potential therapeutic strategies to modulate Treg-mediated immunosuppression in the TME.

## 1 Introduction

Bladder cancer (BC) is the second most common urinary malignancy worldwide (Comperat et al., 2022). Its heterogeneity and complexity are determined by clinical phenotype and etiology (Dyrskjot et al., 2023). Surgical resection is a curative strategy for non-advanced BC, whereas multimodal therapies, including chemotherapy, immunotherapy, and radiotherapy, are used for advanced BC (Powles et al., 2024). Unlike many other cancers, BC is highly immunogenic, and immunotherapies, including *Bacillus* Calmette–Guérin (BCG) and immune checkpoint blockade therapies, have been shown to be effective (Redelman-Sidi et al., 2014). The tumor microenvironment (TME) of BC has been extensively studied, and the presence of various immune cells shapes immune responses, which either inhibit or promote cancer progression (Joseph and Enting, 2019). Among these immune cells, regulatory T cells (Tregs) promote cancer progression; however, the literature on Tregs in BC remains limited. This review describes the immune microenvironment of BC, with a particular focus on Tregs. Additionally, this article discusses the biological roles of Tregs in BC and highlights various therapeutic approaches targeting tumor-infiltrating Tregs.

## 2 Regulatory T cells in the tumor microenvironment

Tregs control immune responses *in vivo* via unique suppressive functions. Tregs were initially identified as CD4^+^CD25^+^ cells in the murine thymus (Sakaguchi et al., 1995). Forkhead box P3 (FOXP3) is a master regulator of Tregs that endows them with suppressive functions by regulating thymic development (Hori et al., 2003). FOXP3^+^ Tregs are more precisely identified as CD4^+^CD25^+^CD127^low^ T cell populations (Liu et al., 2006). Treg-mediated immune suppression involves multiple mechanisms, including cytokine-mediated suppression, cell–cell contact-dependent suppression, and cytokine deprivation (Schmidt et al., 2012). Recent advances in single-cell analyses, including flow cytometry and sequencing, have identified heterogeneous Treg populations (Zemmour et al., 2018). Tregs are divided into naïve and memory Tregs based on CD45RA and FOXP3 expression (Miyara et al., 2009). Similar to conventional T cells, memory Tregs are further divided into Th1-, Th2-, and Th17-like Tregs (Halim et al., 2017). Treg plasticity enables these cells to adapt their phenotype and function in response to local inflammatory signals. Th2-like and Th17-like Tregs can acquire properties resembling those of conventional Th2 or Th17 cells while retaining FOXP3 expression and regulatory characteristics (Koenen et al., 2008; Voo et al., 2009). However, under specific inflammatory conditions, Tregs may exhibit reduced FOXP3 expression and suppressive function, indicating that Treg plasticity encompasses a spectrum of phenotypic and functional states (Koenen et al., 2008).

To date, most human studies have focused on Tregs isolated from peripheral blood; however, tissue-resident and tumor-infiltrating Tregs exhibit distinct phenotypic, transcriptional, and functional characteristics compared with circulating Tregs (De Simone et al., 2016; Plitas et al., 2016). Tumor-infiltrating Tregs exhibit an activated and highly suppressive phenotype and can upregulate immune-regulatory molecules and chemokine receptors, including immune checkpoint-related molecules and CCR8 (De Simone et al., 2016; Plitas et al., 2016). Transcriptomic analyses have further identified tumor-infiltrating Treg-associated gene signatures, including CCR8, LAYN, and MAGEH1 (De Simone et al., 2016). Notably, tumor-resident Tregs in human breast cancer were potently suppressive and exhibited transcriptional profiles resembling normal tissue-resident Tregs rather than activated circulating Tregs, while further upregulating cytokine and chemokine receptor genes, particularly CCR8 (Plitas et al., 2016). These findings indicate that circulating Tregs may not fully reflect the phenotype or suppressive function of Tregs within the TME. In addition to their involvement in immune regulation, tissue Tregs may contribute to tissue regeneration and the maintenance of barrier integrity under physiological conditions (Mathur et al., 2019). Beyond these physiological roles, Tregs are critical regulators in the TME; however, their roles in the TME vary across cancer types (Sato, 2025). Many cancers, including lung and pancreatic cancers, exhibit unfavorable outcomes, especially in the presence of Treg infiltration. Interestingly, colorectal cancer exhibits favorable outcomes in the presence of Treg infiltration, possibly due to local inflammation against the tumor (Saito et al., 2016). Several studies have reported the possible positive and negative effects of Tregs on tumor immunity; however, their specific roles in the TME remain unclear in most cancers. Importantly, several immune checkpoint blockades, such as programmed death-ligand 1 (PD-L1) blockade, possibly increase tumor-associated Treg infiltration and lead to therapeutic resistance (Van Gulijk et al., 2023). Therefore, further translational studies on the TME, with a focus on Tregs, are critical for developing therapeutic strategies targeting tumor-infiltrating Tregs.

## 3 Pathological and molecular classification of bladder cancer

BC is a clinically and biologically heterogeneous malignancy that originates primarily in the urothelium. Cigarette smoking is the most significant environmental risk factor for BC. A large-scale prospective cohort analysis has revealed that current smokers face a substantially higher risk of BC than never-smokers. Furthermore, smoking accounts for approximately half of the total disease burden in both men and women (Freedman et al., 2011). Occupational exposure is a critical factor in bladder carcinogenesis. Long-term evidence from aromatic amine-exposed cohorts indicates a persistent excess mortality risk, confirming the causal link between industrial carcinogens and urothelial malignancy (Pira et al., 2010).

**FIGURE 1:**
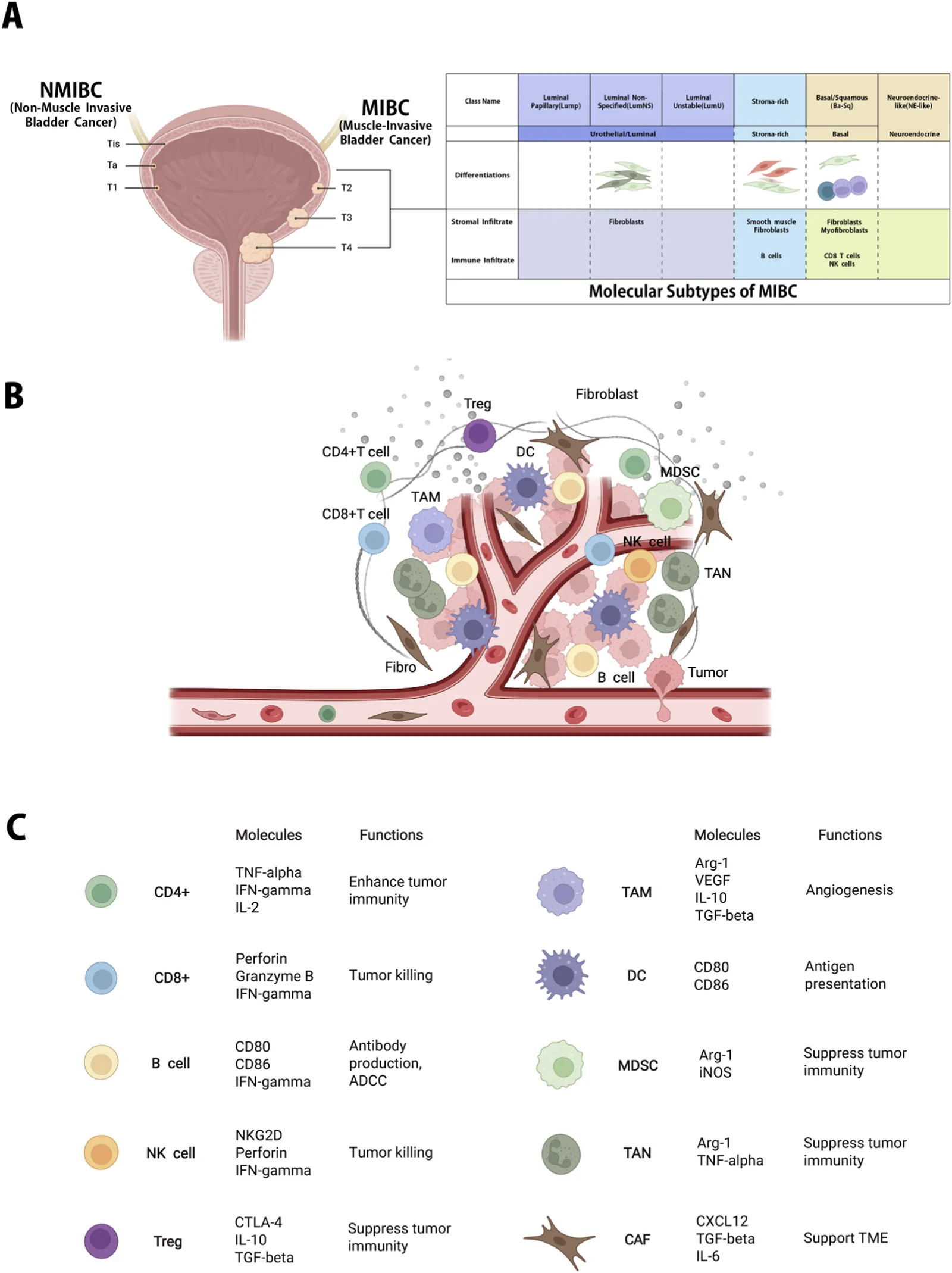
Tumor microenvironment (TME) in bladder cancer (BC). **(A)** BC is staged according to the depth of invasion: non-muscle-invasive BC (NMIBC) encompasses Ta (confined to the urothelium), T1 (invading the lamina propria), and carcinoma *in situ* (CIS), whereas muscle-invasive BC (MIBC) involves invasion into the detrusor muscle. **(B)** The tumor microenvironment is the complex ecosystem surrounding cancer cells. **(C)** Molecular and functional properties of immune cells reside in the TME.

Clinically, BC is staged according to invasion depth: Non-muscle-invasive BC (NMIBC) encompasses Ta (urothelium-confined), T1 (lamina propria-invading), and TIS (carcinoma *in situ*; CIS), whereas muscle-invasive BC (MIBC) involves the detrusor muscle (Figure 1A). A pooled European Organisation for Research and Treatment of Cancer analysis of 2,596 patients with NMIBC has identified tumor count, size, recurrence history, T stage, concomitant CIS, and grade as the primary predictors of clinical outcomes, underscoring the significant biological heterogeneity inherent in this disease (Sylvester et al., 2006). CIS is a flat, high-grade, non-invasive lesion that is clinically important because its presence, particularly when multifocal or concomitant with papillary disease, is associated with an increased risk of disease progression (Pace et al., 2026).

MIBC exhibits significant heterogeneity at the molecular level. A Cancer Genome Atlas analysis has identified various frequently mutated pathways, including cell cycle regulation, chromatin remodeling, and kinase signaling pathways, while also delineating molecular subtypes, such as papillary-like and basal/squamous-like tumors, with potential therapeutic implications (Cancer Genome Atlas Research Network, 2014). A subsequent comprehensive molecular analysis of 412 MIBC cases has further refined the genomic and transcriptomic landscape and demonstrated that molecular subtypes are associated with distinct histological features, mutational patterns, and clinical behaviors (Robertson et al., 2017). The International Consensus Classification later integrated previous molecular classification systems and divided MIBC into six subtypes: Luminal papillary, luminal non-specified, luminal unstable, stroma-rich, basal/squamous, and neuroendocrine-like tumors. These subtypes differ in oncogenic pathways, histological differentiation, and the TME composition, including stromal and immune cell infiltration. Luminal papillary tumors are frequently associated with fibroblast growth factor receptor 3 alterations and papillary morphology and generally exhibit relatively limited immune infiltration compared to basal/squamous or stroma-rich tumors (Kamoun et al., 2020). These findings indicate that BC should be considered not as a single disease but as a spectrum of non-invasive, invasive, flat high-grade, and molecularly defined tumors with distinct biological behaviors.

## 4 Tumor microenvironment of bladder cancer

The BC TME is a dynamic ecosystem composed of malignant urothelial cells, stromal cells, endothelial cells, extracellular matrix components, and diverse immune cell populations (McConkey et al., 2026). Rather than functioning as a passive background, the TME actively shapes tumor growth, invasion, immune escape, and disease progression via reciprocal interactions between malignant and non-malignant cells (Figures 1B,C). Spatial immune profiling of urothelial cancer has shown that tumors differ markedly in immune architecture, including immune-inflamed tumors with abundant immune cell infiltration, immune-excluded tumors with lymphocytes retained in stromal regions, and immune-desert tumors with limited immune cell infiltration (Van Dijk et al., 2021). These spatial patterns suggest that antitumor immunity is shaped not only by immune cell density but also by their specific localization and interactions with distinct tumor and stromal compartments.

Antitumor immunity in the BC TME is mediated in part by cytotoxic lymphocytes and antigen-presenting cells. CD8^+^ T cells can directly eliminate tumor cells, whereas natural killer (NK) cells provide cytotoxic activity and may be particularly relevant to BCG-induced antitumor immunity. BCG stimulation has been shown to activate a cytotoxic CD56^bright^ NK cell population capable of recognizing bladder cancer cells (Esteso et al., 2021). Dendritic cells (DCs), particularly conventional type 1 DCs (cDC1), can link innate and adaptive immune responses by supporting cytotoxic T-cell activation. Single-cell analysis of an experimental BC model has further suggested a functional interaction among NK cells, cDC1, and CD8^+^ T cells. In this model, CD39 inhibition increased intratumoral NK cells, cDC1, and proliferating CD8^+^ T cells while decreasing Treg abundance, and the antitumor effect was impaired by NK-cell depletion or cDC1 deficiency (Liu et al., 2022). Tregs may counteract such antitumor immune networks by suppressing effector T-cell activation and modulating antigen-presenting cell function (Schmidt et al., 2012). Moreover, the BC TME can directly impair NK-cell function; in human BC, TGF-β-rich tumor environments have been shown to reduce NK-cell CD16 expression and impair antibody-dependent cellular cytotoxicity (Wong et al., 2024). Although direct evidence for Treg–NK cell interactions in BC remains limited, the balance between Tregs and cytotoxic lymphocyte–DC networks may contribute to the immune state of the bladder TME.

Myeloid-lineage cells, particularly tumor-associated macrophages (TAMs), act as principal orchestrators of the MIBC immune landscape (Pittet et al., 2022). In MIBC, TAM enrichment is a common feature that correlates with distinct immune cell infiltration patterns and serves as an independent predictor of poor prognosis. Extensive macrophage infiltration reflects a complex TME that oscillates between immunologically active and suppressive states, depending on TAM polarization and the broader immune context, such as programmed death protein 1 (PD-1) and PD-L1 expression (Koll et al., 2023). Myeloid-derived suppressor cells (MDSCs) also contribute to immune suppression in BC. In orthotopic bladder tumor models, MDSCs are enriched in the TME and suppress T cell activity (Bazargan et al., 2023), suggesting that myeloid populations restrain antitumor immune responses. Neutrophils may also participate in BC progression; experimental work has suggested that tumor–neutrophil crosstalk remodels the BC TME and promotes tumor-supportive inflammation (Jing et al., 2024). Stromal components contribute to immune organization and tumor progression. Cancer-associated fibroblasts are increasingly recognized as active regulators of the BC TME rather than simple structural cells. CAF-derived C-X-C motif chemokine ligand 12 promotes immune escape by increasing PD-L1 expression via inhibition of p62-mediated autophagic degradation, and C-X-C motif chemokine ligand 12-rich stromal regions may help form immunosuppressive niches within tumors (Zhang et al., 2023). Tumor-intrinsic pathways are also associated with immune exclusion. In urothelial BC, activation of β-catenin, peroxisome proliferator-activated receptor-γ, and fibroblast growth factor receptor 3 signaling is associated with a non-T cell-inflamed TME (Sweis et al., 2016), suggesting that cancer-cell-intrinsic programs influence whether immune cells enter tumor tissues. Together, these findings indicate that the BC TME is spatially and functionally heterogeneous and is shaped by reciprocal interactions among cytotoxic lymphocytes, Tregs, antigen-presenting and myeloid cells, stromal barriers, and tumor-intrinsic pathways.

## 5 Roles of regulatory T cells in bladder cancer

Tregs are increasingly recognized as important immunosuppressive components in the BC TME. Mechanistically, Tregs can restrain antitumor lymphocyte responses through multiple pathways. CTLA-4-mediated modulation of costimulatory molecules on antigen-presenting cells and IL-2 consumption can limit effector T-cell activation and expansion (Schmidt et al., 2012). Treg-associated membrane-bound TGF-β can suppress NK-cell cytotoxicity and downregulate activating receptors such as NKG2D, although direct evidence for Treg-mediated NK-cell suppression in BC remains limited (Ghiringhelli et al., 2005). In addition, the Treg-associated CD39/CD73 pathway generates extracellular adenosine and contributes to suppression of effector T-cell responses (Deaglio et al., 2007). Notably, recent immunophenotypic analyses of human BC demonstrated that CD39 and CD73 expression on T-cell subsets, including Tregs, was associated with an immunosuppressive immune landscape characterized by increased suppressive myeloid populations and decreased effector T cells (Furriel et al., 2026). These findings suggest that Treg-associated suppressive mechanisms may cooperate with broader immunoregulatory pathways to restrict antitumor T- and NK-cell responses within the BC TME. However, their clinical significance appears to be context-dependent rather than uniform. Increased infiltration of FOXP3-positive T cells, often used as surrogate markers for Tregs, is associated with intravesical recurrence after transurethral resection in NMIBC. A higher proportion of FOXP3-positive T cells among CD3-positive T cells is associated with an increased risk of intravesical recurrence in primary transurethral resection of bladder tumor specimens from patients with NMIBC (Murai et al., 2018). These findings suggest that a Treg-enriched immune environment contributes to local tumor persistence or recurrence in NMIBC. However, FOXP3-positive cell infiltration does not necessarily indicate a uniformly pro-tumoral immune state. Its prognostic value may depend on the balance between suppressive and effector immune cells, tumor stage, and treatment settings. Analyses of tumor-infiltrating lymphocytic subpopulations, including FOXP3-positive cells, have indicated that immune cell composition is associated with clinical outcomes in BC (Horn et al., 2016). Moreover, FOXP3-positive tumor-infiltrating lymphocytes are associated with favorable survival in certain cohorts, whereas FOXP3 expression in tumor cells may have different clinical implications (Winerdal et al., 2011). Therefore, FOXP3 expression should be interpreted within a broader immune context rather than as a simple indicator of immunosuppression. Spatial localization and tissue organization may further influence the clinical effects of Tregs. In bladder CIS treated with intravesical BCG, peritumoral FOXP3-positive cell infiltration beneath the basement membrane is linked to reduced therapeutic efficacy (Fukiage et al., 2025), suggesting that Tregs located near tumor cells exert stronger local immunosuppressive effects. Conversely, FOXP3/tertiary lymphoid structure (TLS)-related parameters, including both FOXP3-positive cells and TLSs, are associated with longer recurrence-free survival in NMIBC, indicating that FOXP3-positive cells should be considered in relation to the surrounding immune architecture rather than merely as markers of immunosuppression (Balcik and Yilmaz, 2025). Treg plasticity may also contribute to the context-dependent roles of Tregs in BC. Tumor-infiltrating Tregs isolated from bladder carcinoma have been shown to acquire IL-17-producing characteristics with reduced FOXP3 expression and suppressive activity under specific *ex vivo* conditions (Chi et al., 2010). These findings suggest that local signals within the BC TME may shape distinct regulatory or Th17-like Treg states, although their relevance *in vivo* remains unclear. Overall, the clinical significance of Tregs in BC depends on tumor stage, treatment context, spatial localization, effector immune cell balance, and organized immune structures, such as TLSs. Therefore, Treg-related biomarkers should be interpreted as part of the broader BC TME rather than as standalone markers of immunosuppression.

## 6 BCG therapy and regulatory T cells

**FIGURE 2:**
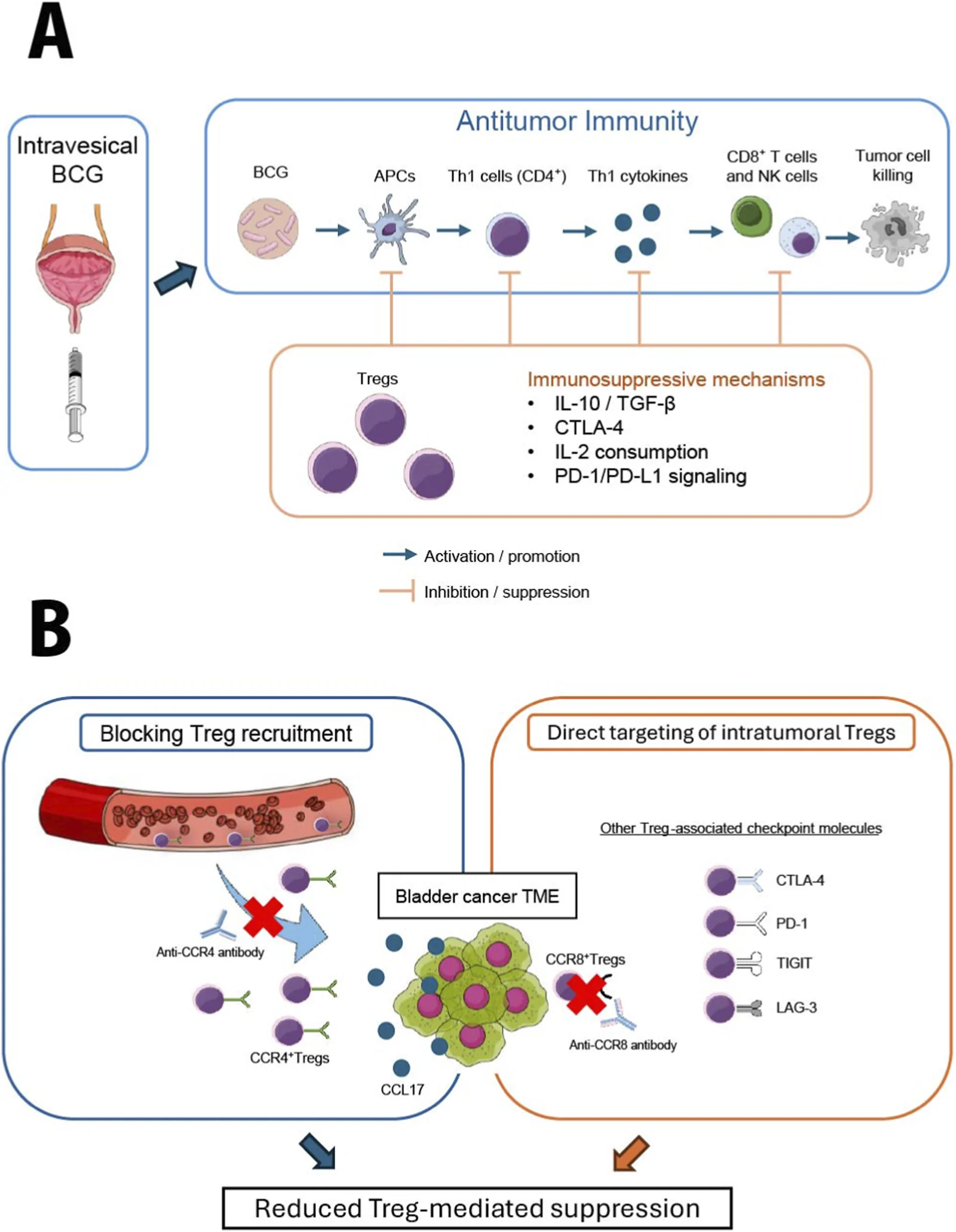
Immunotherapy and Treg-targeted therapy in bladder cancer. **(A)** Mechanism of action of BCG immunotherapy. **(B)** Treg-directed therapies targeting Treg recruitment and co-inhibitory molecules expressed on Tregs.

The clinical relevance of Tregs is particularly high in immunotherapy. BCG remains a standard treatment for high-risk NMIBC, with its antitumor efficacy depending on the coordinated activation of innate immunity, antigen presentation, Th1-polarized CD4^+^ T cell responses, cytotoxic CD8^+^ T cell responses, and natural killer cell activity within the bladder microenvironment (Lim et al., 2020). In this context, Tregs may attenuate BCG-induced antitumor immunity by suppressing effector T cell activation and antigen-presenting cell function via canonical Treg-associated mechanisms, such as the production of immunosuppressive cytokines, cytotoxic T-lymphocyte-associated protein 4-mediated regulation, and interleukin-2 consumption (Figure 2A) (Schmidt et al., 2012). Clinical studies support a link between Treg enrichment and reduced BCG efficacy. Increased infiltration of FOXP3-positive or CD25-positive Treg-related cells into tumor tissues is associated with BCG failure, shorter recurrence-free survival, and shorter progression-free survival (Pichler et al., 2017; Miyake et al., 2017). Consistent with these observations, peritumoral FOXP3-positive cell infiltration in bladder CIS is also associated with reduced therapeutic efficacy of BCG (Fukiage et al., 2025). Beyond pretreatment tissue profiles, BCG therapy may induce counter-regulatory immune responses. Conventional and PD-L1-expressing Tregs are enriched in urine during BCG therapy, and a high urine immunosuppressive score based on these populations is associated with rapid recurrence after treatment (Chevalier et al., 2018). These findings suggest that BCG resistance reflects not only insufficient immune activation but also Treg-centered immune counter-regulation induced or amplified during local immunotherapy. Tregs may also be relevant to immune checkpoint inhibitor (ICI) therapy, although direct evidence linking Tregs to ICI resistance in BC remains limited. The enrichment of PD-L1-expressing Tregs during BCG therapy provides a potential link between Treg-mediated immunosuppression and the PD-1/PD-L1 pathway (Chevalier et al., 2018). BCG-treated tumors can develop adaptive immune resistance, including immune checkpoint-related changes (Kates et al., 2020), suggesting that local immune activation and suppression coexist after intravesical therapy. Therefore, Tregs should be considered as potential contributors to residual immune suppression rather than as established determinants of ICI resistance in BC. These findings suggest that Treg enrichment reduces the efficacy of BCG therapy by suppressing BCG-induced antitumor immunity and participating in compensatory immunoregulatory pathways, including PD-1/PD-L1-related immune suppression. Further studies are necessary to determine whether Treg-related biomarkers predict responses to BCG, ICIs, or combination immunotherapies.

The roles of Tregs in BC should be understood within the broader context of the TME rather than as isolated cellular phenomena. Molecular profiling studies have revealed that urothelial BC exhibits diverse immune states, including tumors with active T cell infiltration and those with limited or excluded T cell infiltration (Sweis et al., 2016). Within this framework, Tregs may contribute to immune suppression as a part of coordinated cellular networks involving tumor cells, myeloid cells, stromal components, and effector lymphocytes. Among these interactions, the relationship between Tregs and myeloid cells appears to be particularly important. In NMIBC treated with intravesical BCG, Treg infiltration is associated with TAMs and interleukin-6-positive tumor cells (Miyake et al., 2017), suggesting that Tregs function within a cytokine- and myeloid cell-rich suppressive microenvironment rather than as an independent immune population. Consistent with this concept, MIBC analyses have suggested that Tregs and TAMs reflect broader immune cell infiltration patterns and may have prognostic relevance (Koll et al., 2023). These findings support the notion that Treg-mediated suppression in BC is shaped by local cellular interactions and inflammatory signals. Recent studies have also highlighted the functional heterogeneity of tumor-infiltrating Tregs. Chemokine receptor axes, including C-C motif chemokine receptor (CCR)-8- and CCR4-related pathways, may explain how Treg phenotype, localization, and recruitment are regulated in the bladder TME (Wang et al., 2020; Maeda et al., 2019). Single-cell and spatial transcriptomic analyses have also revealed substantial intratumoral heterogeneity and complex epithelial–stromal interactions in recurrent BC (Shi et al., 2023), suggesting that immune cell function depends not only on cellular abundance but also on spatial organization and local cellular context. Collectively, these observations suggest that Treg-mediated immune suppression in BC is shaped by cellular networks, spatial organization, and functional heterogeneity in the TME. This complexity also indicates that therapeutic strategies should not simply deplete Tregs broadly but should selectively modulate tumor-infiltrating Treg subsets that sustain local immune suppression.

## 7 Therapeutic implications and future perspectives

Considering their immunosuppressive roles in the BC TME, Tregs represent potential therapeutic targets. However, their systemic depletion can compromise immune tolerance and lead to immune-related toxicities. Therefore, therapeutic strategies should aim to selectively modulate tumor-infiltrating Tregs directly involved in local immune suppression rather than broadly eliminating Tregs in peripheral tissues (Tay et al., 2023). Among potential targets, CCR8 is a promising marker of highly suppressive tumor-infiltrating Tregs. From a therapeutic perspective, CCR8 is appealing as it may facilitate relatively selective targeting of intratumoral Tregs while preserving peripheral Tregs, which are crucial for systemic immune tolerance. Preclinical and translational evidence suggests that CCR8 blockade destabilizes intratumoral Tregs and enhances antitumor immunity in MIBC (Wang et al., 2020), suggesting that CCR8-positive Tregs contribute to maintaining an immunosuppressive niche. Experimental studies in other tumor models have also shown that selective depletion of CCR8-positive tumor-infiltrating Tregs enhances CD8-positive T cell responses and synergizes with PD-1 blockade (Van Damme et al., 2021; Kidani et al., 2022). Additional checkpoint molecules expressed by tumor-infiltrating Tregs, including cytotoxic T-lymphocyte-associated protein 4, PD-1, T cell immunoreceptor with Ig and ITIM domains, and possibly lymphocyte activation gene-3, may also contribute to immunosuppressive signaling; however, their relevance as selective Treg-directed targets in BC remains investigational (Tay et al., 2023). CCR4 may also be a relevant chemokine receptor involved in Treg recruitment in BC. The C-C motif chemokine ligand 17–CCR4 axis promotes Treg accumulation, whereas CCR4 blockade reduces Treg infiltration and prolongs survival in a canine spontaneous BC model. CCR4-positive Tregs have also been observed in human BC tissues, suggesting that this pathway warrants further investigation (Maeda et al., 2019). However, CCR4 is not specific to tumor-infiltrating Tregs and may also be expressed by other T cell subsets. Therefore, CCR4-directed approaches should be considered as potential strategies to modulate Treg recruitment and trafficking rather than as established selective Treg-targeted therapies for human BC. Overall, selective Treg targeting should be viewed as a strategy for remodeling the bladder TME rather than merely depleting Tregs (Figure 2B). Such approaches may be particularly beneficial when combined with therapies that activate antitumor immunity, including BCG and immune checkpoint blockade therapies. Although not designed as a Treg-targeted therapy, a recent phase 3 trial combining BCG therapy with PD-L1 blockade supports the broader concept that modulation of the immunosuppressive bladder TME enhances existing immunotherapeutic strategies (De Santis et al., 2025).

Identifying patients in whom Treg-mediated immune suppression is clinically relevant remains a major challenge. FOXP3-positive cell density alone is unlikely to be sufficient, as FOXP3 expression does not necessarily indicate a suppressive function, and its clinical significance may vary with tumor stage, treatment context, spatial localization, and the surrounding immune context. Tissue-based studies have suggested that FOXP3-positive Treg infiltration, the balance between CD8-positive T and FOXP3-positive cells, and peritumoral FOXP3-positive/CD4-positive ratios exhibit prognostic or predictive value in BC. However, these markers have not yet been prospectively validated for routine clinical use (Murai et al., 2018; Horn et al., 2016; Fukiage et al., 2025). Biomarkers reflecting local immune regulation may be particularly useful in patients undergoing BCG treatment. Urine-based immune monitoring offers a less invasive approach as conventional and PD-L1-expressing Tregs are enriched in urine during BCG therapy and associated with rapid recurrence after treatment (Chevalier et al., 2018). Therefore, dynamic assessments during treatment may provide information beyond pretreatment tissue analysis. Nonetheless, Treg-related markers should be interpreted alongside cytotoxic T cells, myeloid cells, cytokine profiles, and checkpoint-related molecules because Treg accumulation is one component of a broader immunosuppressive network. Future research should move beyond merely describing Treg abundance toward integrated functional and spatial characterization. Single-cell and spatial profiling approaches may help to define clinically relevant Treg subsets and clarify their interactions with tumor cells, stromal cells, macrophages, and effector lymphocytes within distinct niches of the BC TME (Shi et al., 2023). Additionally, targetable phenotypes such as CCR8-expressing intratumoral Tregs may help to link biomarker development to selective Treg-directed therapies (Wang et al., 2020). Ultimately, integrating Treg-based immune markers with clinicopathological and molecular features may guide rational combinations of BCG, immune checkpoint blockade, and selective Treg-targeted therapies.

## 8 Conclusion

Overall, Tregs contribute to immune suppression, tumor progression, and therapeutic resistance in BC. The TME promotes Treg recruitment and stabilization via cytokines, chemokines, metabolic signals, and stromal interactions. Recent single-cell analyses have demonstrated substantial Treg heterogeneity, highlighting the potential of Treg-targeting strategies for BC immunotherapy.
